# Mapping B-Cell Epitopes for Nonspecific Lipid Transfer Proteins of Legumes Consumed in India and Identification of Critical Residues Responsible for IgE Binding

**DOI:** 10.3390/foods10061269

**Published:** 2021-06-02

**Authors:** Ankita Mishra, Ashok Kumar

**Affiliations:** 1 Department of Biological Sciences and Bioengineering, Indian Institute of Technology Kanpur, Kanpur 208016, UP, India; ashokkum@iitk.ac.in; 2 Centre for Environmental Science and Engineering, Indian Institute of Technology Kanpur, Kanpur 208016, UP, India; 3 Centre for Nanosciences, Indian Institute of Technology Kanpur, Kanpur 208016, UP, India; 4 The Mehta Family Centre for Engineering in Medicine, Indian Institute of Technology Kanpur, Kanpur 208016, UP, India

**Keywords:** allergens, b-cell epitopes, legumes, lipid transfer proteins, sequence homology

## Abstract

Nonspecific lipid transfer proteins (nsLTPs) have been categorized as panallergens and display widespread occurrence across plant-kingdom. Present study, investigated B-cell epitopes for LTPs from chickpea, mung-bean, cowpea, pigeon-pea, and soybean via in silico methods. In-silico predicted regions were evaluated for epitope-conservancy and property-based peptide similarity search by different allergen databases. Additionally, the in-silico predicted regions were compared with the experimentally validated epitopes of peach-LTP. Sequence-homology studies showed that chickpea and mung-bean LTPs shared significant homology, i.e., >70% and >60%, respectively, with other LTP allergens from lentil, garden-pea, peanut, etc. Phylogenetic-analysis also showed chickpea and mung-bean LTPs to be closely related to allergenic LTPs from lentil and peanut, respectively. Epitope-conservation analysis showed that two of the predicted B-cell epitopic regions in chickpea and mung-bean LTPs were also conserved in other allergenic LTPs from peach, peanut, garden-pea, lentil, and green-bean, and might serve as conserved B-cell epitopes of the LTP protein family. Property-distance index values for chickpea and mung-bean LTPs also showed that most of the epitopes shared similarity with the reported allergens like-lentil, peanut, apple, plum, tomato, etc. Present findings, may be explored for identification of probable allergenicity of novel LTPs, on the basis of the reported conserved B-cell epitopes, responsible for potential cross-reactivity.

## 1. Introduction

Food allergy is defined as a hypersensitivity reaction induced upon exposure to a particular food allergen (protein) that occurs reproducibly on subsequent exposures. Several characteristics account for allergenicity of a food protein like ability to elicit an immunoglobulin E (IgE) mediated immune response, thermal stability, pepsin resistance, glycosylation, enzymatic activity, etc. [1,2]. However, there exists no thumb rule to differentiate between a ‘food-protein’ and a ‘food-allergen’, since an IgE-mediated immune cascade is a complex interplay between the immune system and the protein in question. However, all allergens (food proteins) on first exposure results in a sensitization phase (no visible symptoms), followed by an effector phase on re-exposure to the same allergen [3,4]. Briefly, sensitization phase involves heavy chain class switching to ε-type and secretion of allergen specific IgEs that bind to the surface of mast cells and basophils. While the re-exposure to the same allergen initiates the effector phase, characterized by cross-linking of the bound IgE(s) and subsequent degranulation with release of pro-inflammatory mediators (accompanied with observable symptoms) [5,6]. Commonly used anti-allergic drugs approved by U.S. ‘food and drug administration’ FDA, include anti-histamines like cetirizine, diphenhydramine, etc. Recently, FDA has approved ‘Palforzia—Aimmune’, i.e., a drug comprising of peanut allergen powder, for specifically treating peanut allergy via oral immunotherapy [7].

IgE-antibodies generated during a type-1 hypersensitivity reaction, in response to an allergen, specifically interacts with the antigen at discrete regions referred as antigenic-determinants or B-cell epitopes [8,9]. Spatial distribution of B-cell epitopes on the surface of proteins, is broadly categorized as linear (sequential/continuous) and/or conformational (nonlinear/discontinuous) epitopes [10,11]. Former, includes amino acids present one after the other in a sequential manner, for example the primary structure of proteins is used as an input feed for predicting linear B-cell epitopes. While the latter, are clusters of amino acids which come in close proximity with each other due to three dimensional (3D)-conformation and account for allergenicity. Proteins naturally exits in the 3D conformation and contribute to allergenicity via conformational epitopes. However, cooking at high temperatures leads to protein denaturation and resulting allergenicity is attributed to linear epitopes [12]. B-cell epitopes are not always unique for an allergen, i.e., some epitopes are partially/fully shared among proteins (allergens). Mostly, species that are phylogenetically related tend to display homologous 3D motifs/folds and cross-reactive epitopes [13,14,15,16]. Nonetheless, sharing of epitopes among unrelated allergens from distant genera has also been reported [17].

Plant LTPs display a ubiquitous presence throughout the plant kingdom and have been divided into two subclasses on the basis of structural organization, i.e., lipid transfer protein 1(LTP1) and lipid transfer protein 2 (LTP2) with a molecular weight of 9 kDa, and 7kDa, respectively [18]. Both these families despite a low sequence similarity (30%) share the general molecular structure. Majority of the plant LTPs possess eight highly conserved cysteine residues and account for thermal stability and resistance to pepsin digestion [19,20]. Consequently, the sensitizing potential of LTP allergens post processing remains intact along with functional allergenic-epitopes. On the contrary, misfolded or unfolded LTP allergens (on thermal treatment or enzymatic digestion) display compromised sensitizing potential and antigenicity due to loss of IgE-binding epitopes.

Due to geographical diversity the dietary consumption of individuals varies among different regions in India. Previous studies, have reported highest prevalence of legume allergy followed by allergy to milk, egg, fish, and eggplant among the Indian population [21,22]. Almost all the legume allergens have been categorized into one or the other of the following protein families and subfamilies, namely, seed storage proteins (cupin and prolamin superfamily), pathogenesis related (PR) proteins, and profilins [23]. Briefly, 7S and 11S globulins (seed storage proteins) belong to the cupin superfamily of proteins, while 2S albumins and ns-LTPs are included in the prolamin superfamily. Further, legume allergens from the PR protein family takes part in defense of the host plant against pathogen attack, whereas profilin protein family include allergens associated with food-pollen allergy [23]. Till date, numerous LTP allergens have been reported from fruits, vegetables, and pollens; however, of all the LTP allergens reported, peach LTP has been considered as the primary IgE elicitor and contributes to clinically relevant cross reactivity among LTP-hypersensitive patients [24,25].

**Figure 1:**
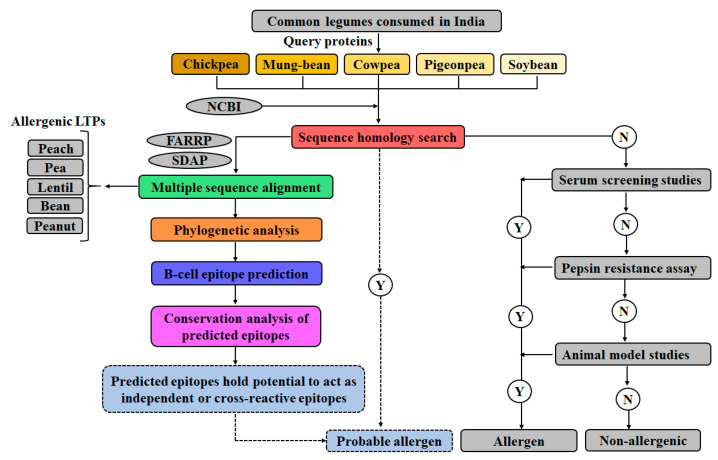
Schematic representation of the workflow employed in the study to evaluate the allergenic potential of five legume LTPs, and mapping of the linear and conformational B-cell epitopes; NCBI (National Center for Biotechnology Information); FARRP (Food Allergy Research and Resource Program); SDAP (Structural Database of Allergenic Proteins); Y (Yes); N (No).

In the present study, allergenic potential of five LTPs from different legumes, namely, chickpea, mung-bean, cowpea, pigeon-pea, and soybean were assessed by sequence homology and phylogenetic analysis, using online web-servers and allergen databases. Further, B-cell epitopes were identified for the LTPs using eight web-servers. In silico predicted epitopes were assessed for ‘epitope-conservancy’ and ‘property-based peptide similarity index (property distance index; PD values) by different tools. B-cell epitopes predicted for the LTPs employed in the study were also compared with the experimentally validated epitopes from peach LTP (Figure 1). Due to extensive cross reactivity reported among the LTP allergens, we considered that large scale screening of the B-cell epitopes from the legumes, and further comparing these regions with the experimentally validated LTP allergens, will provide requisite information essential for delineating conserved B-cell epitopes of LTP allergens (legumes). Present findings, may be used for identification of probable allergenicity of novel LTPs, on the basis of the reported conserved B-cell epitopes.

## 2. Materials and Methods

### 2.1. Sequence Retrieval

Amino acid sequences for five legume LTPs viz. chickpea (CAA05771), mung-bean (CCF23017), cowpea (CAA56113), pigeon-pea (XP_020207090), and soybean (XP_003549896) were retrieved from National Center for Biotechnology Information (NCBI) (www.ncbi.nlm.nih.gov; accessed on 25 January 2021). Further, for comparison with the reported allergenic LTPs, the amino acid sequences for LTPs from peach (AGW21358), garden pea (AJG44053), lentil (AAX35806), green-bean (ADC80502), and peanut (ABX56711) were also retrieved from NCBI.

Multiple sequences were available for LTPs from a single legume crop; however, sequences with ‘lipid transfer protein’ or ‘nonspecific lipid transfer protein’ or ‘lipid transfer like protein’ in the description line of the FASTA format were only selected for the present study. However, selected protein sequences for allergenic LTPs either had ‘lipid transfer protein 1 precursor’ or ‘nonspecific lipid transfer protein 1 precursor’ in the description line of the FASTA format.

### 2.2. Sequence Homology Studies

Involves, comparison of the query protein with the reported allergenic proteins and depending on the percent identity between the two, the potential of a novel protein to act as an allergen is determined [26,27]. In the present study, each of the query protein (i.e., five legume LTPs) was individually compared to each of the member sequences in the allergen databases, namely, Allergenonline (Food Allergy Research and Resource Program, FARRP; http://www.Allergenonline.org/, accessed on 25 January 2021) and Structural Database of Allergenic Proteins (SDAP; http://fermi.utmb.edu/SDAP/, accessed on 25 January 2021), respectively, for prediction of probable allergenicity. Briefly, two search modes were employed in the present study, i.e., ‘full length FASTA alignment’ and ‘80 amino acid sliding window’, respectively. Both the search approaches have previously been described by several groups in details [27]; therefore, in the current manuscript these search modes will not be discussed in detail.

### 2.3. Multiple Sequence Alignment (MSA) and Phylogenetic Analysis

Amino acid sequences retrieved for different LTPs employed in the study were subjected to multiple sequence alignment using the Clustal Omega multiple sequence alignment program (https://www.ebi.ac.uk/Tools/msa/clustalo/, accessed on 25 January 2021). Subsequently, based on the MSA results, the evolutionary relatedness among different legume LTPs was evaluated by constructing a phylogenetic tree (Cladogram or Phylogram), using the neighbor-joining (NJ) algorithm without distance corrections (Clustal Omega).

### 2.4. Mapping Linear and Conformational IgE Binding Epitopes

Seven of the eight webservers employed in the study predicted linear B-cell epitopes, namely, ABCpred [28], BCEPred [29], BepiPred 2.0 [30], LBtope [31], COBEpro [32], SVMTriP [33], and *iBCE-EL* [34], while CBTOPE [35] predicted conformational B-cell epitopes. All the webservers, utilized the primary amino acid sequence of the LTPs as the input feed for prediction studies and were based on the concepts of support vector machines (SVM) or artificial neural networks or hidden Markov model or amino acid propensity scale of hydrophilicity, accessibility, flexibility, antigenicity, etc. For identification of consensus epitopes, preference was given to regions identified by five or more webservers with default settings. Another, point of consideration was that residues predicted by tools having an area under receiver operating characteristic curve (AUC) greater than 0.70 were given priority.

### 2.5. Secondary Structure Prediction

Secondary structures for all the legume LTPs were predicted by the GOR IV method (https://npsa-prabi.ibcp.fr/, accessed on 25 January 2021).

### 2.6. Conservation Analysis of Legume LTPs Employed in the Study

Evolutionary conservation of each amino acid residue for all the five legume LTPs was assessed using the Clustal Omega MSA program (https://www.ebi.ac.uk/Tools/msa/clustalo/, accessed on 25 January 2021). Briefly, each of the query protein, i.e., LTPs from chickpea, pigeon-pea, mung-bean, soybean, and cowpea were individually compared with a set of five allergenic LTPs (from peach, garden pea, lentil, green bean, and peanut) using default settings. Evolutionary scoring was depicted with the help of three distinct signs, i.e., ‘*’ for fully conserved residues, while ‘:’ and ‘.’ for partially conserved residues. Non-conserved residues were devoid of any symbol. The descending order of the evolutionary score was ‘*’ > ‘:’ > ‘.’.

### 2.7. Property-Based Peptide Similarity Index (Property Distance Index—PD Values) of the Predicted Linear Epitopes

Linear epitopes predicted for different legume LTPs were assessed for ‘peptide similarity search (PD Index)’ with the reported allergen sequences in SDAP database. PD index delineates similarity between the query peptide (linear predicted epitope) and other allergen sequences in SDAP database with similar overall physiochemical properties. Peptides with identical sequences display a PD index of 0, while peptides with conservative amino acid substitutions show a lower PD index, usually less than 4. On the contrary, random unrelated peptides display a PD index of more than 10. Therefore, in the present study, the threshold for the PD index was set at < 4, in order to limit less similar matches.

### 2.8. Conservation Analysis of the Residues, Experimentally Validated as B-Cell Epitopes of Peach LTP (Pur p 3), among Fifteen Allergenic LTPs

Amino acid sequences for fifteen allergenic LTPs from different food crops namely, wheat (P24296.2), cabbage (AAA32995.1), celery (E6Y8S8.1), maize (P19656.1), tomato (NP_001316314.1), grape (P80273.2), hazelnut (Q9ATH2.1), walnut (ACI47547.1), sunflower (CAA63340.1), apricot (P81651.2), plum (P82534.1), cherry (Q9M5X8.1), almond (Q43017.1), pear (Q9M5X6.1), and apple (Q9M5X7.1), were retrieved from NCBI. Evolutionary conservation of three experimentally validated B-cell epitopes of Pur p 3, among fifteen allergenic LTPs was assessed using the Clustal Omega MSA program (https://www.ebi.ac.uk/Tools/msa/clustalo/ accessed on e 25 January 2021). Briefly, MSA was performed for all the fifteen LTPs with default settings. Depending on the MSA profile obtained, percent conservation score was calculated for each residue corresponding to the experimentally validated epitopes of Pur p 3. The score was calculated by the formula given below, where ‘X’ corresponds to each residue of the validated epitopes of peach LTP.

(1) $$Conservation\;score\;residue\;‘X’={\frac{Number\;of\;food\;crops\;with\;‘X’\;residue\times 100}{15}}^{}$$

## 3. Results and Discussion

Several experimental approaches like, X-ray crystallography, mutagenesis, phage display, peptide-based assays, nuclear magnetic resonance, deuterium exchange mass spectrometry, etc., delineates information related to B-cell epitopes [36]. However, these methods are time consuming, labor intensive, and require specialized technicians; hence, computational prediction algorithms possess an edge over other methods. Primarily because epitope-prediction webservers/tools are easily accessible, protocol manuals are user friendly, and probability of the predicted epitopes to match with the true epitopes (experimentally validated epitopes) is high. Although, in silico data also comprises of false positives/negatives, but may be explored as an initial screening step, followed by rigorous in vitro experiments.

### 3.1. Sequence Homology Studies (SDAP and FARRP Databases)

Protein sequences for all the five legume LTPs were retrieved from NCBI and subjected to different assessment parameters. Full length FASTA alignment search in both the databases showed, chickpea LTP shared sequence identity >70% with lentil LTP and ≥60% with green bean, peanut, apple, and rubber latex LTPs, respectively (Table 1). Similar, findings were observed for 80 amino acid sliding window search, i.e., query sequence (chickpea LTP) shared >80% identity with lentil LTP, followed by >70% identity with green bean, peanut, apple, and garden pea LTPs respectively. Next, mung-bean LPT was assessed for sequence homology with the reported allergens, both the search parameters, i.e., full length FASTA alignment and 80 amino acid sliding window showed, mung-bean LTP shared >60% identity with LTPs from apple, peanut, and green bean, respectively (Table 1). On the contrary, other legume LTPs from cowpea, pigeon-pea, and soybean shared ≤50% identity with the reported food allergens in SDAP [37] and FARRP [38] databases (Table 1).

The rationale behind use of sequence homology studies for investigating the allergenic potential of a novel protein, is that proteins which share identical stretch of amino acids, may tend to share similar 3D folds/motifs, and may contribute to potential cross reactivity due to the presence of common antigenic determinants. Percent sequence identity is a relative score and there exists no threshold to determine the exact value above which a protein may share cross reactive epitopes. However, previous studies, have reported that greater than 70% sequence identity match corresponds to similarity in the 3D structure of the proteins, and may contribute to potential cross reactivity [39]. As per Food and Agriculture Organization (FAO), United Nations/World Health Organization (FAO/WHO), 2001 decision tree approach [40] or Codex Alimentarius, 2003 [41], sequence homology may be explored as a preliminary test to evaluate the probable allergenicity of a novel protein. Merely using sequence homology may not be adequate in deciphering the allergenic status of a protein; hence, in silico methods may be followed up by in vitro and in vivo experiments [42]. In the present study, as per the sequence homology results, chickpea and mung-bean LTPs might possess potential to act as allergens and may be explored for presence of antigenic determinants.

### 3.2. Multiple Sequence Alignment (MSA) and Phylogenetic Analysis

Phylogenetic analysis elucidated relatedness among different legumes LTPs employed in the study (i.e., LTPs from chickpea, mung-bean, pigeon-pea, cowpea, and soybean) and reported allergenic LTPs from peach, garden pea, lentil, green bean, and peanut. LTPs constitute a well characterized category of food allergens, and some of the examples include Pru p 3 (from peach), Tri a 14 (from wheat), Len c 3 (from lentil), Pis s 3 (from garden pea), Sola l 3 (from tomato), Act c 10 (from kiwi), Mal d 3 (from apple), Pha v 3 (from green bean), Ara h 9 (from peanut), etc [43]. Since, the focus of the study, was mapping IgE binding epitopes of commonly consumed legumes in India; hence, comparisons were performed with the allergenic LTPs from legumes like lentil, garden pea, green bean, peanut and peach were included. Although, peach does not belong to Fabaceae (or Leguminosae) family, yet it was included for the comparative studies, primarily because previous studies have reported that among all the allergenic LTPs, peach LTP has been experimentally validated as the primary elicitor [25]. Implying that if the levels of specific IgE to peach LTP is high, it invariably increases the probability of cross reactivity with other Rosaceae and non-Rosaceae family LTPs [25].

**Figure 2:**
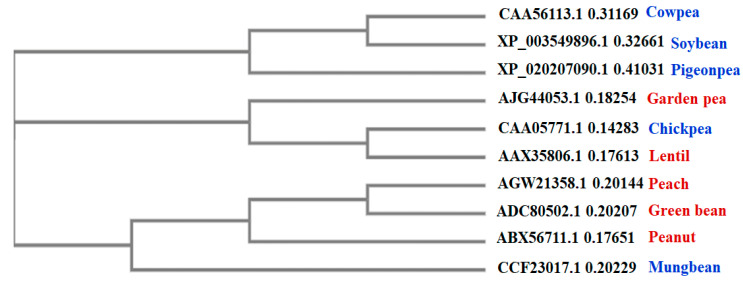
Phylogenetic tree drawn by Clustal Omega MSA tool for evaluating the evolutionary relatedness among LTPs from different legumes and peach. In red are known allergenic LTPs, while in blue are query proteins to be evaluated for potential allergenicity. The tree was drawn by neighbor-joining (NJ) algorithm without distance corrections.

Phylogenetic tree obtained via Clustal Omega showed (Figure 2) chickpea, garden pea and lentil LTPs to be in one clade, followed by peach, green bean, peanut, and mung bean LTPs in another clade, while cowpea, soybean, and pigeon-pea LTPs were grouped in a separate clade (Figure 2). Briefly, the first clade (comprising of cowpea, soybean, and pigeon-pea LTPs) was distant from other allergenic LTPs, suggesting that cowpea, soybean, and pigeon-pea LTPs did not share structural and/or functional characteristics associated with allergenic LTPs. However, in the second clade chickpea LTP was found to be closely related to lentil LTP, followed by garden pea LTP, i.e., chickpea and lentil LTPs shared a common node, signifying close evolutionary relatedness among the two proteins. In the last clade, mung-bean was observed to be more closely related to peanut LTP than peach and green bean LTPs (Figure 2). Hence, the phylogenetic analysis showed that among the five legumes studied, chickpea and mung-bean LTPs were more closely related to the allergenic LTPs. Moreover, this invariably implied that chickpea and mung-bean LTPs, may also share common antigenic determinants with the allergenic LTPs, due to similar structural properties.

### 3.3. Mapping of Consensus Linear and Conformational B-Cell Epitopes

Previous studies have reported, LTPs as panallergens from diverse sources, viz., fruits, vegetables, cereals, pollens, etc. [24,43]. Till date, peach LTP is the most well characterized allergen of the family, with detailed information on experimentally validated epitopes [44], critical IgE binding residues [44], hypoallergenic variants [45], etc. Moreover, all the subsequently reported/investigated LTP allergens have been discussed with reference to peach LTP [46]. In addition, information on the linear/conformational B-cell epitopes of many of the LTP allergens has been determined on the basis of IgE binding with peach positive patients’ sera [47]. That is, experimentally reported epitopes of peach LTP have been utilized as standard for determining potential allergenicity of novel LTPs. Therefore, in the present study, epitope related information was elucidated for five legume LTPs (query proteins), along with prediction of B-cell epitopes for reported allergenic LTPs from peach, garden pea, lentil, green bean and peanut, respectively (Supplementary Table S1). This in turn facilitated, in silico comparison of the predicted B-cell epitopes of query proteins and the reported allergenic LTPs. Further, this also validated the output of the webservers by comparing the predicted epitopes of peach with the experimentally validated epitopes of peach LTP (Supplementary Table S1**)**.

#### 3.3.1. Chickpea LTP

**Figure 3:**
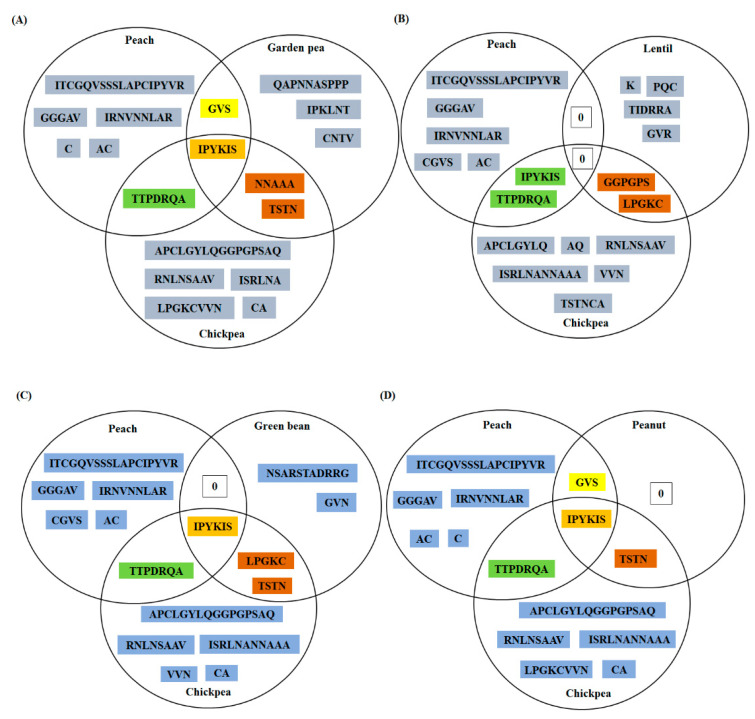
Triads were used for comparing the predicted epitopes of chickpea LTP with the predicted epitopes of peach and other four allergenic legumes. Total four Venn diagrams were generated for chickpea LTP, i.e., chickpea and peach were common to each diagram, only replacement was the allergenic legume LTP, in the order—(**A**) garden pea, (**B**) lentil, (**C**) green bean, and (**D**) peanut, respectively. Only predicted epitopic residues were mentioned in the diagram and ‘0’ denoted absence of any identical regions.

Three B-cell epitopes were predicted for chickpea LTP by majority of the web servers employed in the study (Table 2). First predicted epitope (1st 35–50) was 16 amino acids long and was exclusively a linear epitope, while second (2nd 56–70) and third (3rd 82–112) epitopes were overlapping linear and conformational epitopic regions, respectively (Table 2). Secondary structure prediction for chickpea LPT showed that >60% of the predicted residues were present in the coiled regions (data not shown). Moreover, these residues were more prone to probable interaction with IgE antibodies within an appropriate milieu. Further, predicted epitopes of chickpea LTP were compared to predicted epitopes of peach and other allergenic legumes (Supplementary Figure S1). Each comparison was done using a triad (Venn diagram was constructed) and a total of four triads were generated for chickpea LTP (Figure 3A–D), i.e., chickpea and peach were common to each triad, only replacement was the allergenic legume LTP, in the order—garden pea, lentil, green bean, and peanut, respectively. As shown in Figure 3, ‘I-P-Y-K-I-S’ was a stretch of six amino acids, shared by all the five LTPs, i.e., peach, chickpea, garden pea, peanut, and green bean. While, ‘T-T-P-D-R-Q-A’ was a region common to chickpea and peach LTPs. In addition, it was observed that chickpea in all the four triads shared some specific regions with more than one allergenic legume LTP, like ‘T-S-T-N’, was a common epitopic region among garden pea, chickpea, green bean, and peanut LTPs, respectively. While ‘L-P-G-K-C’ was an epitopic region shared by three LTPs, namely, chickpea, lentil, and green bean, respectively (Figure 3). These findings are suggestive of the fact that predicted epitopes of chickpea LTP share considerably high similarity with the reported allergenic LTPs and may contribute to cross reactivity, if not primary sensitization in atopic/sensitized individuals.

#### 3.3.2. Mung-Bean LTP

**Figure 4:**
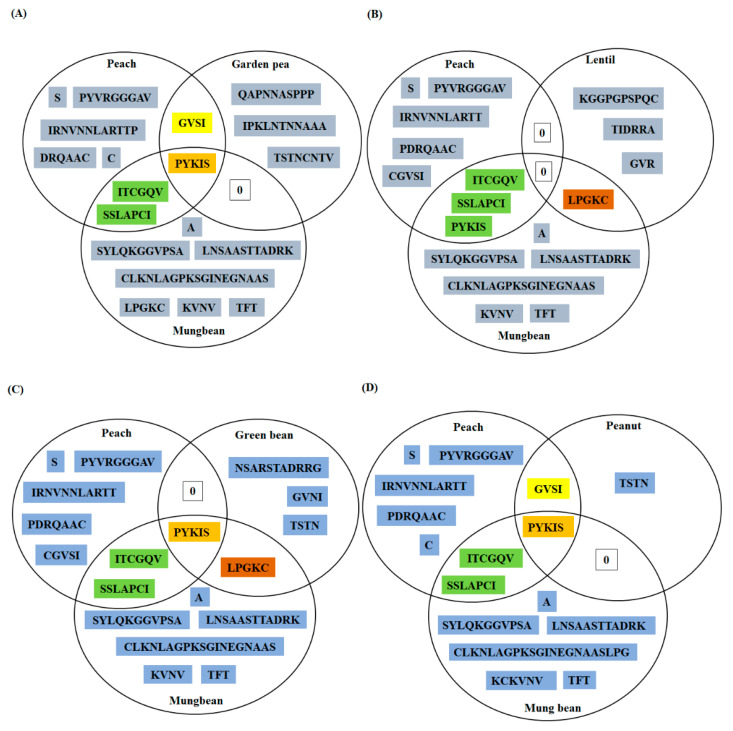
Triads were used for comparing the predicted epitopes of mung-bean LTP with the predicted epitopes of peach and other four allergenic legumes. Total four Venn diagrams were generated for mung-bean LTP, i.e., mung-bean and peach were common to each diagram, only replacement was the allergenic legume LTP, in the order—(**A**) garden pea, (**B**) lentil, (**C**) green bean, and (**D**) peanut, respectively. Only predicted epitopic residues were mentioned in the diagram and ‘0’ denoted absence of any identical regions.

Three B-cell epitopes were predicted for mung-bean LTP and all the three were overlapping linear and conformational epitopes (1st 26–50; 2nd 59–70; and 3rd 75–110) (Table 2). Around 70% of the predicted epitopic residues in mung-bean LTP were present in the coil regions and only a small fraction of the predicted residues contributed to alpha helix and extended strand formation, respectively (data not shown). As previously described, similar triads were constructed for mung-bean LTP for comparison with the predicted epitopes of the allergenic LTPs (Supplementary Figure S1; Figure 4A–D). ‘P-Y-K-I-S’ was a stretch of five amino acids and was identified to be a common to five of the six LTPs employed in the triad, namely, peach, mung bean, garden pea, green bean, and peanut (chickpea included). However, lentil LTP was an exception and lacked this conserved region of five amino acids. Next, ‘I-T-C-G-Q-V’ and ‘S-S-L-A-P-C-I’ were two regions of the predicted B-cell epitopes, shared by both peach and mung-bean LTPs. While, ‘L-P-G-K-C’ was a common epitopic region of five residues shared by three of the LTPs employed in the study namely, lentil, mung bean and green bean (Figure 4).

Presence of identical epitopes/or some regions of the identical epitopes, among two related or distinct proteins contributes to potential cross reactivity [48]. Although several other factors also contribute to clinically relevant cross reactivity like, nature of the identical antigenic determinant, strength of the antigen–antibody interaction, specificity of the antibody, etc. Hence, the in silico findings may be considered as a preliminary indication of the probable allergenicity associated with mung bean LTP and require validation via serum screening studies, etc.

#### 3.3.3. Cowpea LTP

Five B-cell epitopes were predicted for cowpea LTP by majority of the webservers. As like chickpea LTP, the first predicted stretch (1st 29–35) was entirely a linear B-cell epitope, while rest of the predicted regions were overlapping linear and conformational epitopes (2nd 37–50; 3rd 53–65; 4th 72–81; and 5th 93–99), respectively (Table 2). Among the predicted epitopes 72% of the residues were predicted to be involved in coil formation, while rest of the regions were responsible for extended strand synthesis (data not shown).

#### 3.3.4. Pigeon-Pea LTP

Total four epitopes were predicted for pigeon-pea LTP, of these the first predicted region (1st 25–39) was specifically a linear B-cell epitope, while other predicted residues constituted overlapping linear and conformational B-cell epitopes (2nd 47–55; 3rd 69–79; and 4th 124–131), respectively (Table 2). Most of the predicted regions (~80%) were involved in random coil formation as observed by GOR4 software and rest of the epitopic regions were part of the extended strands (data not shown).

#### 3.3.5. Soybean LTP

Out of five epitopes predicted for soybean LTP (1st 3–10; 2nd 21–33; 3rd 46–51; 4th 59–66; and 5th 108–120), the last predicted stretch was a linear epitope, while other four predicted regions were overlapping linear and conformational epitopes (Table 2). Similar to other LTPs employed in the study, maximum number of the predicted residues in soybean LTP were also involved in coil formation (~77%) (data not shown).

As like, chickpea and mung-bean LTPs, triads were attempted for cowpea, pigeon-pea, and soybean LTPs, respectively (Supplementary Figure S2). However, due to lack of identical and/or overlapping epitopes among cowpea, pigeon-pea and soybean LTPs and the allergenic LTPs, the triads could not be generated.

Moreover, it is important to mention that three experimentally validated epitopes of peach LTP, viz., epitope 1st (11–25; APCIPYVRGGGAVPP), epitope 2nd (31–45; IRNVNNLARTTTPDRQ), and epitope 3rd (71–80; GKCGVSIPYK) [44], corroborated with the in silico predicted epitopes of peach LTP in the present study (Supplementary Table S1). Furthermore, epitopic regions shared by different LTPs as mentioned in the chickpea and mung bean triads, respectively, were also present in the experimentally validated epitopes of peach. For instance, two predicted sequences viz. ‘T-T-P-D-R-Q’ and ‘I-P-Y-K’ mentioned in the intersection regions of the chickpea triad, were also present in the second and third experimentally validated epitopes of the peach LTP, respectively [44]. Emphasizing on the fact that predicted epitopes of chickpea and mung-bean LTPs might act as potential epitopes and may share cross reactivity with known LTP allergens.

### 3.4. Conservation Analysis of Individual Amino Acid Residues in Different LTPs

Among all the query proteins studied, maximum number of evolutionary conserved amino acid residues was observed for chickpea LTP, followed by mung-bean, pigeon-pea, cowpea, and soybean, respectively. Around 31 residues were fully conserved in chickpea LTP with reference to the allergenic LTPs, and 27 residues showed conservation with a score of >0.5 in the Gonnet point accepted mutation (PAM) 250 matrix. This implied that these 27 residues were mostly conserved, and if any variation existed, then they were replaced with amino acids having strongly similar properties to the original residues (Supplementary Figure S1). Further, the conservation scores for different residues were utilized for evaluating the conservation pattern of the cross-reactive epitopes.

On overall analysis of the chickpea LTP sequence, four regions were identified as identical across LTPs from different allergenic sources, these were ‘T-T-P-D-R-Q-A’, ‘L-P-G-K-C’, ‘I-P-Y-K-I-S’, and ‘T-S-T-N’. Depending on the wide spread presence of these regions among the predicted epitopes of LTPs, these regions may contribute to potential cross reactivity and may also account for conserved B-cell epitopes of LTPs. It was observed that in ‘T-T-P-D-R-Q-A’ except for ‘P’ and ‘A’ all the residues were partially or fully conserved, suggesting that this predicted B-cell epitope may also be referred as an conserved B-cell epitope of LTPs and may be explored for evaluating potential allergenicity of novel LTPs. Next, all the residues in ‘L-P-G-K-C’ and ‘T-S-T-N’ were fully conserved, except for ‘K’ in former and ‘T’ in latter, both of which displayed partial conservation. Lastly, in ‘I-P-Y-K-I-S’ all the residues were fully conserved, except I and K, former displayed partial conservation, while latter was not conserved. Therefore, these regions may be categorized as conserved B-cell epitopes of legume LTPs or as conserved residues in the LTP allergen family as a whole (Supplementary Figure S1).

In mung-bean LTP, 30 residues were fully conserved, and 24 of the residues were conserved with a score of >0.5 in the Gonnet PAM 250 matrix (Supplementary Figure S1). Three regions among the predicted B-cell epitopes of mung-bean LTP, and also shared by other allergenic legumes were ‘I-T-C-G-Q-V’, ‘S-S-L-A-P-C-I’, and ‘P-Y-K-I-S’. These regions were assessed for presence of evolutionarily conserved residues. In ‘I-T-C-G-Q-V’ all the residues were partially or fully conserved except for ‘Q’, while in ‘S-S-L-A-P-C-I’ two residues, i.e., ‘P’ and ‘C’ were fully conserved, while ‘L’ and ‘I’ were partially conserved, and other residues did not show any sort of conservation. Lastly, in ‘P-Y-K-I-S’ as like before in case of chickpea LTP, four residues displayed full conservation, except, K with no conservation at all. These findings provide insights for the use of these sequences as conserved B-cell residues, common to the reported allergenic LTPs and query proteins. Further, these residues may be explored for clinically relevant cross-reactivity by designing peptides and studying relevant IgE binding with LTP positive patient sera.

Total number of fully conserved residues in cowpea, pigeon-pea and soybean were 11, 14, and 10, respectively. While the count for the partially conserved residues were 15, 14, and 19, in the same order as above. Since, no specific regions present in the predicted epitopes of these three legume LTPs, were shared by the allergenic LTPs, therefore, the conservation of the epitopic regions was not analyzed for these three legume LTPs (Supplementary Figure S2).

### 3.5. Peptide Similarity Search of Predicted Linear Epitopes (PD Index)

As previously described in Section 2.7, the PD index threshold was set at < 4 for the present study. Three linear epitopes predicted for chickpea LTP shared similar physicochemical properties with the sequences available in SDAP. These sequences were identified by calculating property distance (PD) values for each of the match. Sequences with a PD value of less than four suggested high similarity and were included in the study. As shown in Supplementary Table S2, sequences which shared similarity with the B-cell epitopes of chickpea LTP, belonged to different allergen sources such as wheat (Tri a 14), lentil (Len c 3), green bean (Pha v 3), peach (Pru p 3), apple (Mal d 3), etc.

Similarly, for mung-bean LTPs, the predicted linear epitopes were subjected to a peptide similarity search in SDAP database and only those allergenic sequences were selected which displayed a PD index of less than four. The allergenic sequences which displayed similarity with mung bean predicted linear epitopes (Supplementary Table S2), were from maize (Zea m 14), strawberry (Fra a 3), peanut (Ara h 9), etc. Further, for all the other legume LTPs studied, i.e., cowpea, pigeon-pea, and soybean, linear predicted epitopes showed similarity with the allergenic sequences reported in SDAP database, with PD index > 4. Hence, these matches were not included in the study, since the PD index was higher than the selected threshold. The rationale behind selecting a low PD-index was to include only those hits, which corroborated to significant similarity between the query proteins (predicted epitopes) and the allergenic sequences. In addition, it also inhibited inclusion of false positive and false negative results. Among all the LTPs studied significant matches were observed for chickpea and mung-bean LTP predicted epitopes.

### 3.6. Conservation Analysis of the Residues, Experimentally Validated as B-Cell Epitopes of Peach LTP (Pur p 3), among Fifteen Allergenic LTPs

**Figure 5:**
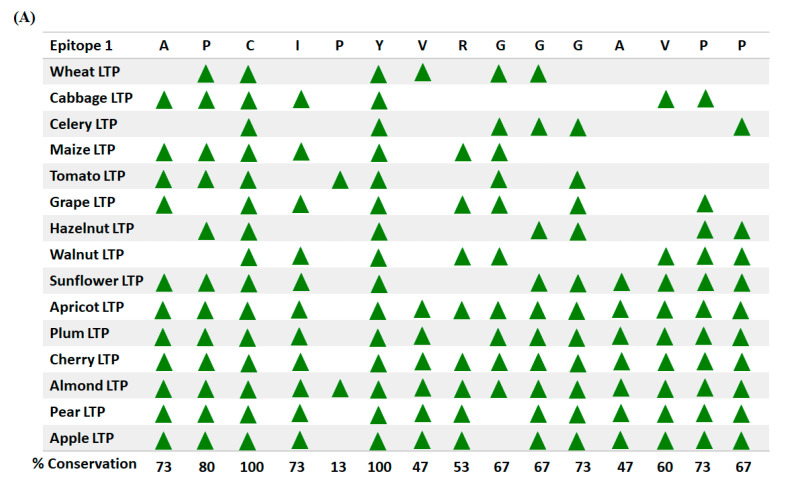

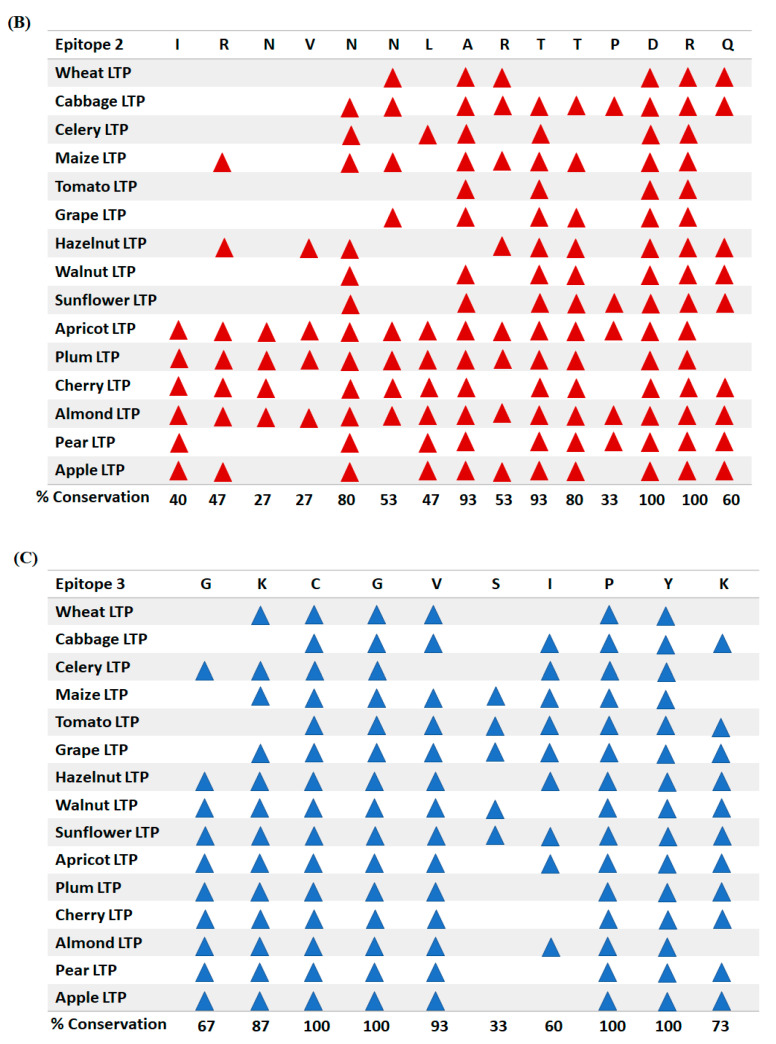
Multiple sequence alignment (MSA) of fifteen allergenic LTPs, in order to identify conservation score for residues corresponding to the experimentally validated B-cell epitopes of peach LTP. (**A**): Epitope 1st, (**B**): Epitope 2nd, and (**C**): Epitope 3rd, respectively.

Till date several studies, and significant amount of clinical data has already validated the antigenic dominance of peach LTPs among other LTP allergens [49]. Therefore, investigating the evolutionarily conserved pattern of the B-cell epitopes of Pur p 3 among other LTP allergens will provide insights into the conserved B-cell epitopes of the LTP allergen family. Further, these regions may be considered as highlights for predicting probable allergenicity or lack of it among novel LTPs. As shown in Figure 5A–C, two residues in epitope 1st [11–25; APCIPYVRGGGAVPP], i.e., ‘C’(position-13) and ‘Y’(position-16); two residues in epitope 2nd [31–45; IRNVNNLARTTTPDRQ], i.e., ‘D’(position-43) and ‘R’(position-44); and four residues in epitope 3rd [71–80; GKCGVSIPYK], i.e., ‘C’(position-73), ‘G’(position-74), ‘P’(position-78), and ‘Y’(position-79) displayed a conservation score of 100 among 15 allergenic LTPs, while other residues displayed mixed scores in the range of 93% to 13%. These findings corroborate with Figure 3; Figure 4 of the present study, where a five amino acid long predicted epitopic region, i.e., P-Y-K-I-S, has been identified as common to peach, garden pea, peanut, green bean, chickpea, and mung bean. Moreover, some other residues as reported in Figure 5 show greater than 90% conservation score and have also been identified in chickpea and mung bean LTPs. Therefore, among the legume LTPs studied chickpea and mung bean LTPs display requisite potential to act as cross-reactive allergens, and may be further explored by in vitro serum screening studies.

Furthermore, previous studies have reported the allergenic potential of chickpea and mung bean LTPs via sequence homology and multiple sequence alignment studies [50,51]. However, in-depth analysis of the linear/conformational epitopes, information related to the conserved B-cell epitopic residues, PD similarity values, exact match peptides, etc., is not available. Therefore, the present study was accomplished with an aim to draft a detailed source of epitope related information for five legume LTPs, namely, chickpea, mung-bean, cowpea, pigeon-pea, and soybean.

LTP hypersensitivity in patients is diverse and complex; therefore, a generalization cannot be deduced on the basis of few studies with limited number of LTP positive patient samples. For instance, ELISA inhibition of kiwi LTP with Pur p 3 positive serum does not render complete abolishment of the IgE binding of the former, implying that kiwi LTP display some unique antigenic determinants not recognized by Pur p 3 specific IgE’s [52]. Similarly, Tri a 14 (wheat) and Pur p 3 (peach) LTP allergens share only few common B-cell epitopes, i.e., IgEs from patients with baker’s asthma mask only few epitopic sites of the Pur p 3 protein and do not abrogate significant IgE binding [53]. These observations imply that, several factors account for cross reactivity among proteins, but the possibility of structurally homologous proteins to share cross reactive epitopes is significantly high. Tri a 14 and Pur p 3 both are LTPs and belong to the same family of proteins; however, they share < 50% identity, and account for reduced cross-reactivity among the B-cell epitopes. Although, there are recent advancements in the research protocols employed for evaluating cross reactivity among proteins, nonetheless sequence-homology based studies for evaluating clinically relevant cross-reactivity, still remains relevant and authentic [54].

## 4. Conclusions

With the increase in the diversity of the food proteins being consumed, i.e., exploration of new food sources as alternative protein supplements for the human body, the burden on the food safety testing protocols is constantly increasing. Therefore, under such circumstances an ideal scenario would be to assimilate as much information as possible for all known and related food proteins. So that the information obtained may be utilized for evaluating potential allergenicity of new/novel food proteins. Consequently, in silico studies were explored as a preliminary testing approach, in the present study, for investigating the potential allergenicity associated with five legumes LTPs. Along-with sequence homology studies several other in silico tools/methods were explored for evaluating structural similarities among the allergenic LTPs and the query proteins. Depending on the results obtained in the present study (from all the in silico characterization studies), it was inferred that chickpea and mung-bean LTPs displayed significant homology, phylogenetic relatedness, high epitope-conservancy, and antigenicity with the reported allergenic LTPs, and might act as potential allergens.

## Figures and Tables

**Table 1:** LTP sequences from chickpea, mung-bean, cowpea, pigeon-pea and soybean shared sequence homology with the allergens listed in two databases namely—FARRP and SDAP.

Sequence Homology Studies for Chickpea LTP Using FARRP Database			
**S. no.**	**Allergen Source**	**Full Length FASTA Alignment**	**80 Amino Acid Sliding Window**
		**Identity Percentage**	**Identity Percentage**
1	*Lens culinaris*	71.80%	81.30%
2	*Pisum sativum*	70.60%	76.20%
3	*Phaseolus vulgaris*	62.70%	72.80%
4	*Malus x domestica*	60.30%	70.40%
5	*Arachis hypogaea*	67.00%	70.00%
6	*Prunus armeniaca*	57.40%	68.80%
7	*Hevea brasiliensis*	59.80%	66.70%
**Sequence Homology Studies for Chickpea LTP Using SDAP Database**			
**S. no.**	**Allergen Source**	**Full Length FASTA Alignment**	**80 Amino Acid Sliding Window**
		**Identity Percentage**	**Identity Percentage**
1	*Lens culinaris*	71.55%	81.25%
2	*Phaseolus vulgaris*	63.79%	--
3	*Hevea brasiliensis*	60.34%	--
4	*Arachis hypogaea*	62.07%	--
5	*Malus x domestica*	59.48%	--
**Sequence Homology Studies for Mungbean LTP Using FARRP Database**			
**S. no.**	**Allergen Source**	**Full Length FASTA Alignment**	**80 Amino Acid Sliding Window**
		**Identity Percentage**	**Identity Percentage**
1	*Malus x domestica*	62.10%	68.80%
2	*Arachis hypogaea*	67.00%	67.55%
3	*Phaseolus vulgaris*	62.40%	63.70%
4	*Fragaria x ananassa*	57.90%	62.50%
5	*Lens culinaris*	55.80%	61.30%
**Sequence Homology Studies for Mungbean LTP Using SDAP Database**			
**S. no.**	**Allergen source**	**Full Length FASTA Alignment**	**80 Amino Acid Sliding Window**
		**Identity Percentage**	**Identity Percentage**
1	*Phaseolus vulgaris*	62.93%	62.50%
2	*Malus x domestica*	61.21%	67.50%
3	*Arachis hypogaea*	62.07%	67.50%
4	*Lens culinaris*	54.31%	--
**Sequence Homology Studies for Cowpea LTP Using FARRP Database**			
**S. no.**	**Allergen Source**	**Full Length FASTA Alignment**	**80 Amino Acid Sliding Window**
		**Identity Percentage**	**Identity Percentage**
1	*Lens culinaris*	29.9%	--
2	*Solanum lycopersicum*	50%	50%
**Sequence Homology Studies for Cowpea LTP Using SDAP Database**			
**S. no.**	**Allergen Source**	**Full Length FASTA Alignment**	**80 Amino Acid Sliding Window**
		**Identity Percentage**	**Identity Percentage**
1	*Lens culinaris*	32.32%	33.75%
**Sequence Homology Studies for Pigeon-pea LTP Using FARRP Database**			
**S. no.**	**Allergen source**	**Full Length FASTA Alignment**	**80 Amino Acid Sliding Window**
		**Identity Percentage**	**Identity Percentage**
1	*Zea mays*	27%	--
2	*Triticum aestivum*	29.8%	--
**Sequence Homology Studies for Pigeon-pea LTP Using SDAP Database**			
**S. no.**	**Allergen Source**	**Full Length FASTA Alignment**	**80 Amino Acid Sliding Window**
		**Identity Percentage**	**Identity Percentage**
1	*Lens culinaris*	--	31.25%
**Sequence Homology Studies for Soybean LTP Using FARRP Database**			
**S. no.**	**Allergen Source**	**Full Length FASTA Alignment**	**80 Amino Acid Sliding Window**
		**Identity Percentage**	**Identity Percentage**
1	*Solanum lycopersicum*	32.70%	35%
**Sequence Homology Studies for Soybean LTP Using SDAP Database**			
**S. no.**	**Allergen Source**	**Full Length FASTA Alignment**	**80 Amino Acid Sliding Window**
		**Identity Percentage**	**Identity Percentage**
1	*Arachis hypogaea*	23.18%	28.75%
2	*Hevea brasiliensis*	--	36.25%

**Table 2:** Consensus of linear and conformational IgE binding epitopes of chickpea, mung-bean, cowpea, pigeon-pea, and soybean LTPs, respectively, using eight webservers.

LTP Source	No. of Epitopes	Position	Predicted Epitope Sequence	Prediction Servers
**Chickpea**	1	35–50	APCLGYLQGGPGPSAQ	ABCpred BCEPred BepiPred 2.0 LBtope COBEpro SVMTriP *iBCE-EL* CBTOPE
	2	56–70	RNLNSAAVTTPDRQA	
	3	82–112	ISRLNANNAAALPGKCVVNIPYKISTSTNCA	
**Mung-bean**	**No. of epitopes**	**Position**	**Predicted epitope sequence**	
	1	26–50	ITCGQVASSLAPCISYLQKGGVPSA	
	2	59–70	LNSAASTTADRK	
	3	75–110	CLKNLAGPKSGINEGNAASLPGKCKVNVPYKISTFT	
**Cowpea**	**No. of epitopes**	**Position**	**Predicted epitope sequence**	
	1	29–35	AEAVTCN	
	2	37–50	TELSSCVPAITGGS	
	3	53–65	SSTCCSKLKVQEP	
	4	72–81	KNPSLKQYVN	
	5	93–99	GVTYPNC	
**Pigeon-pea**	**No. of epitopes**	**Position**	**Predicted epitope sequence**	
	1	25–39	ASDIPATCNGDEPVL	
	2	47–55	VNKVPNPSS	
	3	69–79	MGDNTGQGIRD	
	4	124–131	LSNQEKNY	
**Soybean**	**No. of epitopes**	**Position**	**Predicted epitope sequence**	
	1	3–10	MGGGCKCL	
	2	21–33	RSLAEAQSGSSTT	
	3	46–51	NGTTTP	
	4	59–66	LKQTVENQ	
	5	108–120	NGSAPAPGSGPPP	

**Note:** Some of the predicted regions are highlighted in yellow as these are exclusively, linear epitopes. For identification of the consensus regions only those residues were selected which were predicted by more than five webservers.

## Supplementary Materials

The following are available online at https://www.mdpi.com/article/10.3390/foods10061269/s1, Figure S1: Multiple sequence alignment (MSA) of the query protein with five allergenic LTPs, fully and partially conserved residues are denoted by ‘*’, ‘:’ & ‘.’ (A) & (B) Chickpea and mung-bean LTPs (accession no.), respectively were highlighted in yellow and the corresponding epitopic regions common to the query protein and the reported allergens were marked in cyan, while the partially conserved residues were marked in magenta, Figure S2: Multiple sequence alignment (MSA) of the query protein with five allergenic LTPs, fully and partially conserved residues were denoted by ‘*’, ‘:’ & ‘.’ (A), (B) & (C) Cowpea, Pigeonpea & Soybean LTPs, respectively (corresponding accession numbers highlighted in yellow), Table S1: Consensus of linear and conformational IgE binding epitopes of five LTP allergens, along with the experimentally validated B-cell epitopes of Peach LTP, Table S2: Peptide similarity search of the predicted epitopes of chickpea and mung-bean LTP with the known allergens in SDAP database. Only those similarity hits of the predicted B-cell epitopes of LTPs with peptides of known allergens, were included which displayed a PD value of less than 4.
