# Supplementary material for: Mapping B-Cell Epitopes for Nonspecific Lipid Transfer Proteins of Legumes Consumed in India and Identification of Critical Residues Responsible for IgE Binding
Source: Foods. 2021 Jun 2;10(6):1269. doi: 10.3390/foods10061269 (PMC8227083; doi:10.3390/foods10061269)
Supplement: Supplementary file 1 [file foods-10-01269-s001.zip › foods-1173737-supplementary.pdf]

Table S1. Consensus of linear and conformational IgE binding epitopes of five LTP allergens, along with the experimentally validated B-cell epitopes of Peach LTP.

**PEACH**			
**Consensus of predicted linear and conformational B-cell epitopes of Peach LTP**			
**S. No.**	**Predicted B-cell epitopes**	**Position**	**Prediction servers**
1	ITCGQVSSSLAPCIPYVRGGGAV	28-50	ABCpred, BCEPred, BepiPred 2.0, LBtope, COBEpro, SVMTriP, *iBCE-EL and* CBTOPE
2	IRNVNNLARTTPDRQAAC	58-75	
3	CGVSIPYKIS	100-109	
**Experimentally validated B-cell epitopes of Peach LTP, García-Casado et al., 2003**			
1	APCIPYVRGGGAVPP	11-25	Validated via. IgE binding studies
2	IRNVNNLARTTPDRQ	31-45	
3	GKCGVSIPYK	71-80	

**GARDEN PEA**			
**Consensus of predicted linear and conformational B-cell epitopes of garden pea LTP**			
**S. No.**	**Predicted B-cell epitopes**	**Position**	**Prediction servers**
1	QAPNNASPPP	44-53	ABCpred, BCEPred, BepiPred 2.0, LBtope, COBEpro, SVMTriP, *iBCE-EL and* CBTOPE
2	IPKLNTNNAAA	86-96	
3	GVSIPYKISTSTNCNTV	102-118	

**LENTIL**			
**Consensus of predicted linear and conformational B-cell epitopes of lentil LTP**			
**S. No.**	**Predicted B-cell epitopes**	**Position**	**Prediction servers**
1	KGGPGPSPQC	44-53	ABCpred, BCEPred, BepiPred 2.0, LBtope, COBEpro, SVMTriP, *iBCE-EL and* CBTOPE
2	TIDRRA	67-72	
3	LPGKCGVR	95-102	

**GREEN BEAN**			
**Consensus of predicted linear and conformational B-cell epitopes of green bean LTP**			
**S. No.**	**Predicted B-cell epitopes**	**Position**	**Prediction servers**
1	NSARSTADRRG	60-70	ABCpred, BCEPred, BepiPred 2.0, LBtope, COBEpro, SVMTriP, *iBCE-EL and* CBTOPE
2	LPGKCGVNIPYKISTSTN	93-110	

**PEANUT**			
**Consensus of predicted linear and conformational B-cell epitopes of peanut LTP**			
**S. No.**	**Predicted B-cell epitopes**	**Position**	**Prediction servers**
1	GVSIPYKISTSTN	98-110	ABCpred, BCEPred, BepiPred 2.0, LBtope, COBEpro, SVMTriP, *iBCE-EL and* CBTOPE

Table S2. Peptide similarity search of the predicted epitopes of chickpea and mung-bean LTP with the known allergens in SDAP database. Only those similarity hits of the predicted B-cell epitopes of LTPs with peptides of known allergens, were included which displayed a PD value of less than 4.

**Chickpea LTP**				
**Epitope 1: APCLGYLQGGPGPSAQ**				
**Allergen**	***PD* Sequence Similarity Index**	**Start Residue**	**Matching region**	**End Residue**
Wheat Tri a 14	3.78	11	RPCLSYVQGGPGPSGQ	26

Allergen	*PD* Sequence Similarity Index	Start Residue	Matching region	End Residue
Lentil Len c 3	3.95	37	SPCLTYLTGGPGPSPQ	52
**Epitope 2: RNLNSAAVTTPDRQA**				
**Allergen**	***PD* Sequence Similarity Index**	**Start Residue**	**Matching region**	**End Residue**
Green Bean Pha v 3	3.34	58	RSLNAAAKTTPDRQA	72
Peach Pru p 3	3.73	32	RNVNNLARTTPDRQA	46
Strawberry Fra a 3	3.84	58	RSLNSAAKTTADRQA	72
**Epitope 3: ISRLNANNAAALPGKCVVNIPYKISTSTNC**				
**Allergen**	***PD* Sequence Similarity Index**	**Start Residue**	**Matching region**	**End Residue**
Lentil Len c 3	1.99	84	ITKLNTNNAAALPGKCGVNIPYKISTTTNC	113
Apricot Pru ar 3	3.28	58	ISGVNPNNAAALPGKCGVNIPYKISASTNC	87
Cherry Pru av 3	3.42	84	VPGVNANNAAALPGKCGVNVPYKISPSTNC	113
Apple Mal d 3	3.64	82	ISGVNPNNAAGLPGKCGVNVPYKISTSTNC	111
Green bean Pha v 3	3.84	82	VRGLNPNNAQALPGKCGVNIPYKISTSTNC	111
Plum Pru d 3	3.92	58	IPGVNPNNAAALPGKCGVNVPYKISASTNC	87

**Mung-bean LTP**				
**Epitope 1: ITCGQVASSLAPCISYLQKGGVPSA**				
**Allergen**	***PD* Sequence Similarity Index**	**Start Residue**	**Matching region**	**End Residue**
Mulberry Mor n 3	3.70	1	ITCGQVSSSLAPCINYLRAGGVVPA	25
Peanut Ara h 9	3.85	1	LSCGQVNSALAPCITFLTKGGVPSG	25
**Epitope 2: LNSAASTTADRK**				
**Allergen**	***PD* Sequence Similarity Index**	**Start Residue**	**Matching region**	**End Residue**
Strawberry Fra a 3	2.14	60	LNSAAKTTADRQ	71
Maize Zea m 14	2.72	63	LNNAARTTADRR	74
Mulberry Mor n 3	2.79	34	LNNAAKTTADRQ	45
Rubber latex Hev b 12	3.32	59	INNAAKTTADRR	70
**Epitope 3: CLKNLAGPKSGINEGNAASLPGKCKVNVPY**				
**Allergen**	***PD* Sequence Similarity Index**	**Start Residue**	**Matching region**	**End Residue**
Pear Pyr c 3	3.74	74	CLKNLAGSVSGVNPGNAESLPGKCGVNVPY	103
Apple Mal d 3	3.83	74	CLKNLAGSISGVNPNNAAGLPGKCGVNVPY	103

**Cowpea LTP**				
**Epitope 1: AEAVTCN**				
**Allergen**	***PD* Sequence Similarity Index**	**Start Residue**	**Matching region**	**End Residue**
Wheat Tri a 18	3.55	200	AEAITAN	206
Pear Pyr c 3	3.84	22	AHAITCS	28

(A)

```
AJG44053.1     --MARSMKLACVALVICMVV-IAPMAEAALSCGTVSADMAPCVTYLQAPNNASPPPPCCA 57
CAA05771.1     ---MASMKVVCVALIMCIV--IAPMAESAITCGRVDTALAPCLGYLQGG--PGPSAQCCG 53
AAX35806.1     ---MASLRVSCLVALMCMVVISAPMAEAAISCGTVSGALVPCLTYLKGG--PGPSPQCCG 55
AGW21358.1     MAYSAMTKLALVV-ALCMV-VSVPIAQ-AITCGQVSSSLAPCIPYVRGG--GAVPPACCN 55
ADC80502.1     ---MASVKFACVV-VLCMVVVGAHTAQ-GMTCGQVQSNLVPCVTFLQNG--GFVPAGCCN 53
ABX56711.1     ---MASLKFAFVM-LVCMAMVGAPMVN-AISCGQVNSALAPCIPFLTKG--GAPPPACCS 53
                       :.  :   :*:.   .  .: .::** *.  :.**: ::            **

AJG44053.1     GVKKLLAAATTTPDRQAACNCLKSAAGSIPKLNTNNAAALPGKCGVSIPYKISTSTNCNT 117
CAA05771.1     GVRNLNSAAVTTPDRQAACNCLKSAAGSISRLNANNAAALPGKCVVNIPYKISTSTNCAT 113
AAX35806.1     GVKRLNGAARTTIDRRAACNCLKSSAGSISGLKPGNVATLPGKCGVRLPYTISTSTNCNT 115
AGW21358.1     GIRNVNNLARTTPDRQAACNCLKQLSASVPGVNPNNAAALPGKCGVSIPYKISASTNCAT 115
ADC80502.1     GVRNIMNSARSTADRRGICNCLKTAAGAVRGLNPNNAQALPGKCGVNIPYKISTSTNCAS 113
ABX56711.1     GVRGLLGALRTTADRQAACNCLKAAAGSLRGLNQGNAAALPGRCGVSIPYKISTSTNCAT 113
               *::  :      :* **:  *****  :.::  :: .*. :***:*  * :**.**:****  :

AJG44053.1     VRF       120
CAA05771.1     IRV       116
AAX35806.1     IRF       118
AGW21358.1     VK-       117
ADC80502.1     IN-       115
ABX56711.1     IKF       116
               :.
```

(B)

```
AJG44053.1     --MARSMKLACVALVICMVV-IAPMAEAALSCGTVSADMAPCVTYLQAPNNASPPPPCCA 57
AAX35806.1     ---MASLRVSCLVALMCMVVISAPMAEAAISCGTVSGALVPCLTYLKGG--PGPSPQCCG 55
AGW21358.1     MAYSAMTKLALVV-ALCM-VVSVPIAQ-AITCGQVSSSLAPCIPYVRGG--GAVPPACCN 55
CCF23017.1     ---MASLKVACMVAVVFMVVVSAHMAH-AITCGQVASSLAPCISYLQKG--GVPSASCCS 54
ADC80502.1     ---MASVKFACVV-VLCMVVVGAHTAQ-GMTCGQVQSNLVPCVTFLQNG--GFVPAGCCN 53
ABX56711.1     ---MASLKFAFVM-LVCMAMVGAPMVN-AISCGQVNSALAPCIPFLTKG--GAPPPACCS 53
                       :.:  :   :  *  :   .  .. .::** *. :.**: ::            **

AJG44053.1     GVKKLLAAATTTPDRQAACNCLKSAAGSIPKLNTNNAAALPGKCGVSIPYKISTSTNCNT 117
AAX35806.1     GVKRLNGAARTTIDRRAACNCLKSSAGSISGLKPGNVATLPGKCGVRLPYTISTSTNCNT 115
AGW21358.1     GIRNVNNLARTTPDRQAACNCLKQLSASVPGVNPNNAAALPGKCGVSIPYKISASTNCAT 115
CCF23017.1     GVKALNSAASTTADRKTACNCLKNLAGPKSGINEGNAASLPGKCKVNVPYKISTFTNCAN 114
ADC80502.1     GVRNIMNSARSTADRRGICNCLKTAAGAVRGLNPNNAQALPGKCGVNIPYKISTSTNCAS 113
ABX56711.1     GVRGLLGALRTTADRQAACNCLKAAAGSLRGLNQGNAAALPGRCGVSIPYKISTSTNCAT 113
               *::  :      :* **:  *****  :.    :: .*. :***:*  * :**.**:  ***  .

AJG44053.1     VRF       120
AAX35806.1     IRF       118
AGW21358.1     VK-       117
CCF23017.1     IK-       116
ADC80502.1     IN-       115
ABX56711.1     IKF       116
               :.
```

Figure S1. Multiple sequence alignment (MSA) of the query protein with five allergenic LTPs, fully and partially conserved residues are denoted by '*', ':' & '.' (A) & (B) Chickpea and mung-bean LTPs (accession no.), respectively were highlighted in yellow and the corresponding epitopic regions common to the query protein and the reported allergens were marked in cyan, while the partially conserved residues were marked in magenta.

(A)

```
CAA56113.1      MTMKMKMKMSVVCAVVVVALFLIDVGPVAE-AVTCNP--TELSSCVPAITGG--SKPSST 55
AJG44053.1      ----MARSMKLACVALVICMVV-I-APMAEAALSCGTVSADMAPCVTYLQAPNNASPPPP 54
AAX35806.1      -----MASLRVSCLVALMCMVVIS-APMAEAAISCGTVSGALVPCLTYLKGG--PGPSPQ 52
AGW21358.1      --MAYSAMTKLALVV-ALCMV-VS-VPIAQ-AITCGQVSSSLAPCIPYVRGG--GAVPPA 52
ADC80502.1      -----MASVKFACVV-VLCMVVVG-AHTAQ-GMTCGQVQSNLVPCVTFLQNG--GFVPAG 50
ABX56711.1      -----MASLKFAFVM-LVCMAMVG-APMVN-AISCGQVNSALAPCIPFLTKG--GAPPPA 50
                          .      :.:        .: .::*.     :  *:  :

CAA56113.1      CCSKLKVQ---------EPCLCNYIKNPSLK-QYVNSPGAKKVLSNCGVTYPNC------ 99
AJG44053.1      CCAGVKKLLAAATTTPDRQAACNCLKSAAGSIPKLNTNNAAALPGKCGVSIPYKISTSTN 114
AAX35806.1      CCGGVKRLNGAARTTIDRRAACNCLKSSAGSISGLKPGNVATLPGKCGVRLPYTISTSTN 112
AGW21358.1      CCNGIRNVNNLARTTPDRQAACNCLKQLSASVPGVNPNNAAALPGKCGVSIPYKISASTN 112
ADC80502.1      CCNGVRNIMNSARSTADRRGICNCLKTAAGAVRGLNPNNAQALPGKCGVNIPYKISTSTN 110
ABX56711.1      CCSGVRGLLGALRTTADRQAACNCLKAAAGSLRGLNQGNAAALPGRCGVSIPYKISTSTN 110
                **  ::           .   ** :*  :     ::  ..  : ..***  *

CAA56113.1      ------    99
AJG44053.1      CNTVRF    120
AAX35806.1      CNTIRF    118
AGW21358.1      CATVK-    117
ADC80502.1      CASIN-    115
ABX56711.1      CATIKF    116
```

(B)

```
XP_020207090.1      MGEKKVLGLVMFVMAYTLAMITHTASDIPATCNGDEPVLTFCGPYLVNK-VPNPSSDCCK    59
AJG44053.1          --MARSMKLACVALVICMVV-IAPMAEAALSCGTVSADMAPCVTYLQAPNNASPPPPCCA    57
AAX35806.1          ---MASLRVSCLVALMCMVVISAPMAEAAISCGTVSGALVPCLTYLKGG--PGPSPQCCG    55
AGW21358.1          MAYSAMTKLALVV-ALCMV-VSVPIAQ-AITCGQVSSSLAPCIPYVRGG--GAVPPACCN    55
ADC80502.1          ---MASVKFACVV-VLCMVVVGAHTAQ-GMTCGQVQSNLVPCVTFLQNG--GFVPAGCCN    53
ABX56711.1          ---MASLKFAFVM-LVCMAMVGAPMVN-AISCGQVNSALAPCIPFLTKG--GAPPPACCS    53
                            .  .     :.       :   :*.  .  :. *  ::           **

XP_020207090.1      GATKVFNRAMGDNTGQGIRDLCNCLRGAGP-SLGFQQPNLINLPSLCGIKTTFSMPLCIL    118
AJG44053.1          GVKKLLAAATT---TPDRQAACNCLKSAAGSIPKLNTNNAAALPGKCGVSIPYKISTSTN    114
AAX35806.1          GVKRLNGAART---TIDRRAACNCLKSSAGSISGLKPGNVATLPGKCGVRLPYTISTSTN    112
AGW21358.1          GIRNVNNLART---TPDRQAACNCLKQLSASVPGVNPNNAAALPGKCGVSIPYKISASTN    112
ADC80502.1          GVRNIMNSARS---TADRRGICNCLKTAAGAVRGLNPNNAQALPGKCGVNIPYKISTSTN    110
ABX56711.1          GVRGLLGALRT---TADRQAACNCLKAAAGSLRGLNQGNAAALPGRCGVSIPYKISTSTN    110
                    *   :           . :  ****:  .     .:  *   **. **:   :.:  .

XP_020207090.1      GPNVLLSNQEKNY 131
AJG44053.1          CNTVRF------- 120
AAX35806.1          CNTIRF------- 118
AGW21358.1          CATVK-------- 117
ADC80502.1          CASIN-------- 115
ABX56711.1          CATIKF------- 116
                      .:
```

(C)

```
XP_003549896.1      MKMGG--GCKCLVSLVLALVLMRSLAEAQSGSSTTCAQELIPCVNFLNGT--TTPPSSCC    56
AJG44053.1          --MARSMKLACVALVICMVV-IAPMAEA-ALSCGTVSADMAPCVTYLQAPNNASPPPPCC    56
AAX35806.1          ---MASLRVSCLVALMCMVVISAPMAEA-AISCGTVSGALVPCLTYLKGG--PGPSPQCC    54
AGW21358.1          MAYSAMTKLALVV-ALCMV-VSVPIAQ--AITCGQVSSSLAPCIPYVRGG--GAVPPACC    54
ADC80502.1          ---MASVKFACVV-VLCMVVVGAHTAQ--GMTCGQVQSNLVPCVTFLQNG--GFVPAGCC    52
ABX56711.1          ---MASLKFAFVM-LVCMAMVGAPMVN--AISCGQVNSALAPCIPFLTKG--GAPPPACC    52
                               :   :         .:  . :.      : **: ::           **

XP_003549896.1      DPLKQTV-------ENQLDCLCNIFFSPGLLQSFNVSVDQALALSRRCGVTNGITSCTNG    109
AJG44053.1          AGVKKLLAAATTTPDRQAACNCLKSA-AGSIPK--LNTNNAAALPGKCGVSIPYKISTST    113
AAX35806.1          GGVKRLNGAARTTIDRRAACNCLKSS-AGSISG--LKPGNVATLPGKCGVRLPYTISTST    111
AGW21358.1          NGIRNVNNLARTTPDRQAACNCLKQL-SASVPG--VNPNNAAALPGKCGVSIPYKISAST    111
ADC80502.1          NGVRNIMNSARSTADRRGICNCLKTA-AGAVRG--LNPNNAQALPGKCGVNIPYKISTST    109
ABX56711.1          SGVRGLLGALRTTADRQAACNCLKAA-AGSLRG--LNQGNAAALPGRCGVSIPYKISTST    109
                      ::          :.:  * *      . :    :. .:. :*  :***    . .:.

XP_003549896.1      SAPAPGSGPPPVTPGGDKGGAGRVTFTGLSFLLLFWVSMLFN    151
AJG44053.1          NC-------------------NTVRF----------------    120
AAX35806.1          NC-------------------NTIRF----------------    118
AGW21358.1          NC-------------------ATVK-----------------    117
ADC80502.1          NC-------------------ASIN-----------------    115
ABX56711.1          NC-------------------ATIKF----------------    116
                    ..                     :
```

Figure S2: Multiple sequence alignment (MSA) of the query protein with five allergenic LTPs, fully and partially conserved residues were denoted by ‘*’, ‘:’ & ‘.’ (A), (B) & (C) Cowpea, Pigeonpea & Soybean LTPs, respectively (corresponding accession numbers highlighted in yellow).
